# Antiproliferative activity and apoptosis-inducing mechanism of constituents from *Toona sinensis* on human cancer cells

**DOI:** 10.1186/1475-2867-13-12

**Published:** 2013-02-09

**Authors:** Shengjie Yang, Qi Zhao, Hongmei Xiang, Minjie Liu, Qiuyun Zhang, Wei Xue, Baoan Song, Song Yang

**Affiliations:** 1 State-Local Joint Laboratory for Comprehensive Utilization of Biomass, State Key Laboratory Breeding Base of Green Pesticide and Agricultural Bioengineering, Key Laboratory of Green Pesticide and Agricultural Bioengineering, Ministry of Education, Guizhou University, Guiyang, 550025, P.R. China; 2 Ctr for R&D of Fine Chemicals, Guizhou University, Huaxi St, Guiyang, 550025, China

**Keywords:** *Toona sinensis*, Antiproliferation, Apoptosis, Pathway

## Abstract

**Background:**

Natural products, including plants, microorganisms and marines, have been considered as valuable sources for anticancer drug discovery. Many Chinese herbs have been discovered to be potential sources of antitumor drugs.

**Methods:**

In the present study, we investigated the antitumor efficacy of the compounds isolated from *Toona sinensis*, an important herbal medicine. The inhibitory activities of these compounds were investigated on MGC-803, PC3, A549, MCF-7, and NIH3T3 cells *in vitro* by MTT assay. The mechanism of the antitumor action of active compounds was investigated through AO/EB staining, Hoechst 33258 staining, TUNEL assay, flow cytometry analysis, and western blotting analysis.

**Results:**

Fifteen compounds were isolated from the roots of *Toona sinensis*. Betulonic acid (BTA) and 3-oxours-12-en-28-oic acid (OEA) isolated from the plant inhibited the proliferation of MGC-803 and PC3 cells, with IC_50_ values of 17.7 *μ*M and 13.6 *μ*M, 26.5 *μ*M and 21.9 *μ*M, respectively. Both could lead to cell apoptosis, and apoptosis ratios reached 27.3% and 24.5% in MGC-803 cells at 72 h after treatment at 20 *μ*M, respectively. Moreover, the study of cancer cell apoptotic signaling pathway indicated that both of them could induce cancer cell apoptosis through the mitochondrial pathway, involving the expressions of p53, Bax, caspase 9 and caspase 3.

**Conclusions:**

The study shows that most of the compounds obtained from *Toona sinensis* could inhibit the growth of human cancer cells. Furthermore, BTA and OEA exhibited potent antitumor activities *via* induction of cancer cell apoptosis.

## Background

Among the conventional antitumor cytotoxic chemotherapies, many compounds are derived from natural products [1-3]. Over 60% of the current anticancer drugs have their origin in one way or another from natural sources [4,5]. Natural compounds had attracted considerable attention as cancer chemopreventive agents and also as cancer therapeutics [6,7]. As cancer cells have evolved multiple mechanisms to resist the induction of programmed cell death (apoptosis), the modulation of apoptosis signaling pathways by natural compounds have been demonstrated to constitute a key event in these antitumor activities [8,9]. *Toona sinensis*, an important herb medicine, belongs to the Meliaceae family which comprises approximately 50 genera and 1400 species throughout the world [10], and is widely distributed in China except Xinjiang and Inner Mongolia Autonomous Regions. The objective of present study was to evaluate the potency of the components from the plant for growth inhibiting of human cancer cell lines and to study their antitumor mechanism. Fifteen compounds were isolated from the plant, and these compounds were bioassayed on human gastric cancer cell line MGC-803, prostatic cancer cell line PC3, lung cancer cell line A549, breast cancer cell line MCF-7, and mouse embryonic fibroblast cell line NIH3T3 *in vitro* by MTT assay. Interestingly, it was found that betulonic acid (BTA) and 3-oxours-12-en-28-oic acid (OEA) had the potent inhibitory activities against MGC-803 and PC3 cell lines, and were less toxic on normal cells than on the investigated cancer cell lines. Also, BTA and OEA are betulinic acid (BA) and ursolic acid (UA) derivatives, respectively. BA and UA are naturally occurring pentacyclic triterpenoids which are widely distributed in the plant kingdom [11,12]. It was found that BA could inhibit growth of cancer cells [13,14], without affecting normal cells [15,16], and it was a highly selective growth inhibitor of human melanoma, neuroectodermal and malignant tumor cells [17]. UA has also been reported to show significant cytotoxicity against some tumor cell lines [13,18-21]. There are a few reports on the anticancer effects of BTA and OEA on various tumor cells recently. Some studies have shown that BTA could inhibit the growth of various types of human tumor cell lines, including SGC-7901, HepG-2 [22], LNCaP, and DU-145 [23] cells. In 1999, Min et al. found that OEA possessed antitumor activity on A549, SK-OV-3, SK-MEL-2, XF498, and HCT15 cells, with low IC_50_ values (< 5 *μ*g/mL) [18]. However, no report was found on the antitumor mechanism of the two compounds. Thus, the mechanism of action needs to be further clarified. Further investigation of BTA and OEA was carried out on MGC-803 and PC3 cells, and experimental results of fluorescent staining and flow cytometry analysis indicated that the two compounds could induce cell apoptosis. In addition, the mechanism underlying apoptosis of BTA and OEA was also investigated in this study. To the best of our knowledge, this is the first report on apoptosis inducing of BTA and OEA in MGC-803 and PC3 cells.

## Methods

### Plant material

Fresh samples of *Toona sinensis* were collected from Bijie, Guizhou Province in China, in August 2011. Prof. Qingde Long, Department of Medicine, Guiyang Medical University, identified the plant material. A voucher specimen was deposited at Guiyang Medical University, Guiyang, China.

### Cell culture

MGC-803, PC3, A549. MCF-7, and NIH3T3 cell lines were obtained from the Institute of Biochemistry and Cell Biology, China Academy of Science. MGC-803 is human gastric cancer cell line, PC3 is prostatic cancer cell line, A549 is lung cancer cell line, MCF-7 is breast cancer cell line, and NIH3T3 is mouse embryonic fibroblast cell line. The entire cancer cell lines were maintained in the RPMI 1640 medium and NIH3T3 was maintained in the DMEM medium. They were supplemented with 10% heat-inactivated fetal bovine serum (FBS) in a humidified atmosphere of 5% CO_2_ at 37°C. All cell lines were maintained at 37°C in a humidified 5% carbon dioxide and 95% air incubator.

### MTT assays

The antitumor activities of the compounds were determined by MTT assay. All tested compounds were dissolved in DMSO and subsequently diluted in the culture medium before treatment of the cultured cells. When the cells were 80-90% confluent, they were harvested by treatment with a solution containing 0.25% trypsin, thoroughly washed and resuspended in supplemented growth medium. Cells (1×10^4^/well) were plated in 100 *μ*L of medium/well in 96-well plate. After incubations overnight, the cells were treated with different concentrations of extracts or compounds for 72 h. Thereafter, 100 *μ*L of MTT (Beyotime Co., Jiangsu, China) solution was added to each well and then incubated for 4 h. The colored MTT-formazan crystals which were produced from MTT were dissolved in SDS for 12 h. And then the OD values were measured at 595 nm with a microplate reader (BIO-RAD, model 680), which is directly proportional to the number of living cells in culture [24-26].

### AO/EB staining

The active compounds were investigated for apoptotic activity by AO/EB staining. When the cells were 80-90% confluent, they were harvested by treatment with a solution containing 0.25% trypsin, thoroughly washed and resuspended in supplemented growth medium. The cells were seeded in 6-well tissue culture plates (5×10^4^ cell/mL, 0.6 mL/well). After incubations overnight, the medium was removed and replaced with fresh medium plus 10% FBS and then supplemented with compounds (20 *μ*mol/L). After the treatment period, 20 *μ*L of the AO/EB dye mix (Beyotime Co., Shanghai, China) were added to each well, and the apoptotic cells were viewed and counted under the fluorescent microscope (OLYMPUS Co., Tokyo Met, Japan) [27,28].

### Hoechst 33258 staining

Morphological assessment of apoptotic cells was performed using Hoechst 33258 staining method. The cells were seeded in 6-well tissue culture plates (5×10^4^ cell/mL, 0.6 mL/well). After incubations overnight, the medium was removed and replaced with fresh medium plus 10% FBS and then supplemented with compounds (20 *μ*mol/L) for a certain range of treatment time. The culture medium containing compounds was removed, and the cells were fixed in 4% paraformaldehyde for 10 min. The cells were washed twice with PBS, and were consequently stained with 0.5 mL of Hoechst 33258 staining (Beyotime Co., Jiangsu, China) for 5 min. The stained nuclei were washed twice with PBS, and were consequently observed under an IX71SIF-3 fluorescence microscope at 350 nm excitation and 460 nm emissions [29].

### TUNEL assay

The cells (5×10^4^ cell/mL, 0.6 mL/well) were seeded in 6-well tissue culture plates. Following incubation, the medium was removed and replaced with fresh medium plus 10% FBS and then supplemented with compounds (20 *μ*mol/L). TUNEL assays were performed using a colorimetric TUNEL apoptosis assay kit according to the manufacturer’s instructions. (1) After the treatment period, cells were washed with 1×PBS and fixed in 4% paraformaldehyde for 40 min. The cells were washed once with PBS, and were consequently permeabilized with immunol staining wash buffer for 2 min on ice. (2) The cells were rewashed once with PBS, and were consequently incubated in 0.3% H_2_O_2_ in methanol at room temperature for 20 min to inactivate the endogenous peroxidases, after which the cells were washed thrice with PBS. (3) The cells were incubated with 2 *μ*L of TdT-enzyme and 48 *μ*L of Biotin-dUTP per specimen for 60 min at 37°C. The cells were terminated for 10 min, and were consequently incubated with streptavidin-HRP (50 *μ*L per specimen) conjugate diluted at 1:50 in sample diluent for 30 min. (4) The cells were washed three times with PBS, and were consequently incubated with diaminobenzidine solution (200 *μ*L per specimen) for 10 min. At last, the cells were rewashed twice with PBS, and were consequently imaged under an XDS-1B inverted biological microscope [30].

### Flow cytometry analysis

Prepared MGC-803 cells (1×10^6^/mL) were washed twice with cold PBS and then re-suspended gently in 500 *μ*L binding buffer. Thereafter, cells were stained in 5 *μ*L Annexin V-FITC and shaked well. Finally, 5 *μ*L PI was added to these cells and incubated for 20 min in a dark place, analyzed by FACS Calibur, Becton Dickinson [31,32].

### Caspase 3 enzyme assay

Cells were collected after treatment with BTA and OEA at 2.5, 5, and 10 *μ*M for 12 h, respectively. Prepared MGC-803 cells (1×10^6^/mL, 5 ml) were washed twice with cold PBS. Then, 100 *μ*L of lysis buffer was added to the cells for 25 min on ice and centrifuged at 16000 g for 15 min. 80 *μ*L of reaction buffer and 10 *μ*L of Ac-DEVED-*p*NA were added to 10 *μ*L of supernatant liquid. After incubating at 37°C for 2–3 h in darkness, the absorbance was measured at 405 nm, with the lysis buffer and reaction buffer as control

### Caspase 9 enzyme assay

Cells were collected after treatment with BTA and OEA at 2.5, 5, and 10 *μ*M for 12 h, respectively. Prepared MGC-803 cells (1×10^6^/mL, 5 ml) were washed twice with cold PBS. Then, 100 *μ*L of lysis buffer was added to the cells for 25 min on ice and centrifuged at 16000 g for 15 min. 80 *μ*L of reaction buffer and 10 *μ*L of Ac-LEHD-pNA were added to 10 *μ*L of supernatant liquid. After incubating at 37°C for 2–3 h in darkness, the absorbance was measured at 405 nm, with the lysis buffer and reaction buffer as control.

### Western botting analysis

Cells were collected after treatment with BTA and OEA at 2.5, 5, and 10 *μ*M for 12 h, respectively. Western blotting analysis was performed as previously described [33], using the following antibodies at dilutions of 1:500 to 1:1000: anti-p53, anti-Bax, and anti-*β* actin (Cell signaling technology, Beverly, MA).

### Statistical analysis

All statistical analyses were performed using SPSS 10.0, and the data were analyzed using one-way ANOVA. The mean separations were performed using the least significant difference method. Each experiment was performed in triplicate, and all experiments were run thrice and yielded similar results. Measurements from all the replicates were combined, and the treatment effects were analyzed.

## Results and discussion

The roots of *Toona sinensis* collected from Guizhou province were studied, and fifteen compounds were isolated from the plants. The extraction and purification process of the compounds from the plant and their NMR data are presented in Additional file 1.

The potential effect of the compounds from *Toona sinensis* was investigated on the viability of MGC-803, PC3, A549, MCF-7, and NIH3T3 cells by MTT assay, with ADM (Adriamycin) being used as the positive control and culture medium containing 0.1% DMSO used as the negative control. The inhibitory percentage of cancer cells was treated with 20 *μ*mol/L of each compound for 72 h. The results are summarized in Table 1. It could be seen from Table 1 that both of BTA and OEA showed potent antitumor activities against MGC-803 and PC3 cell lines. The inhibitory ratios of BTA and OEA at 72 h after treatment were 56.1% and 45.2% against MGC-803 cells, 63.4% and 42.5% against PC3 cells, 22.1% and 23.6% against NIH3T3 normal cell line, respectively. In addition, BTA also had good activities against MCF-7 cells, with inhibitory ratio of 51.2%. Thus, the two compounds were less toxic on normal cells than on the investigated cancer cell lines.

**Table 1:** Antitumor activities of the isolated compounds on the proliferation of different cell lines

**Compound**	**Inhibitory Rate for Different Cell Lines (%, mean ± SD)**^**a**^				
	**MGC-803**	**PC3**	**A549**	**MCF-7**	**NIH3T3**
*β*-sitosterol (**1**)	23.5 ± 2.1	16.5 ± 2.3	12.6 ± 3.1	18.9 ± 1.7	5.6 ± 2.4
*α*-Amyrin (**2**)	23.5 ± 5.4	17.8 ± 4.9	7.8 ± 1.5	10.4 ± 1.8	7.6 ± 4.5
Daucosterol (**3**)	12.3 ± 4.1	9.8 ± 3.6	4.5 ± 1.8	7.2 ± 5.2	4.3 ± 2.3
Quercetin (**4**)	17.2 ± 1.7	22.7 ± 1.4	16.9 ± 2.4	42.2 ± 1.6	4.2 ± 2.5
(+)-Catechin (**5**)	52.1 ± 5.7	49.6 ± 2.3	45.3 ± 3.2	37.6 ± 3.9	28.9 ± 4.3
(−)-Epicatchin (**6**)	45.5 ± 4.1	50.6 ± 1.6	47.1 ± 1.1	43.2 ± 3.6	22.5± 3.8
Kampferol (**7**)	58.2 ± 3.0	46.1 ± 5.9	42.0 ± 2.2	39.2 ± 6.8	11.1 ± 6.7
3-Oxours-12-en-28-oic acid (**8**)	45.2 ± 2.0	42.5 ± 1.4	35.9 ± 0.8	37.2 ± 1.5	23.6 ± 1.3
Ursolic acid (**9**)	52.1 ± 2.2	55.6 ± 2.4	37.6 ± 1.7	47.8 ± 1.2	21.7 ± 4.9
Betulonic acid (**10**)	56.1 ± 2.6	63.4 ± 4.2	35.2 ± 2.4	51.2 ± 4.4	22.1 ± 6.2
Gallic acid (**11**)	55.7± 1. 9	40.2 ± 4.2	46.3 ± 3.6	33.1 ± 1.4	13.6 ± 2.2
Betulinic acid (1**3**)	36.9 ± 1.6	23.6 ± 3.4	33.5 ± 2.3	41.6 ± 3.7	23.4 ± 2.9
ADM	92.1 ± 1.3	93.4 ± 2.6	96.2 ± 0.8	91.1 ± 2.2	99.4 ± 0.4

Note: ^a^ Inhibitory percentage of cells treated with each compound 20 *μ*mol/L for 72 h and SD = standard deviation.

**Figure 1:**
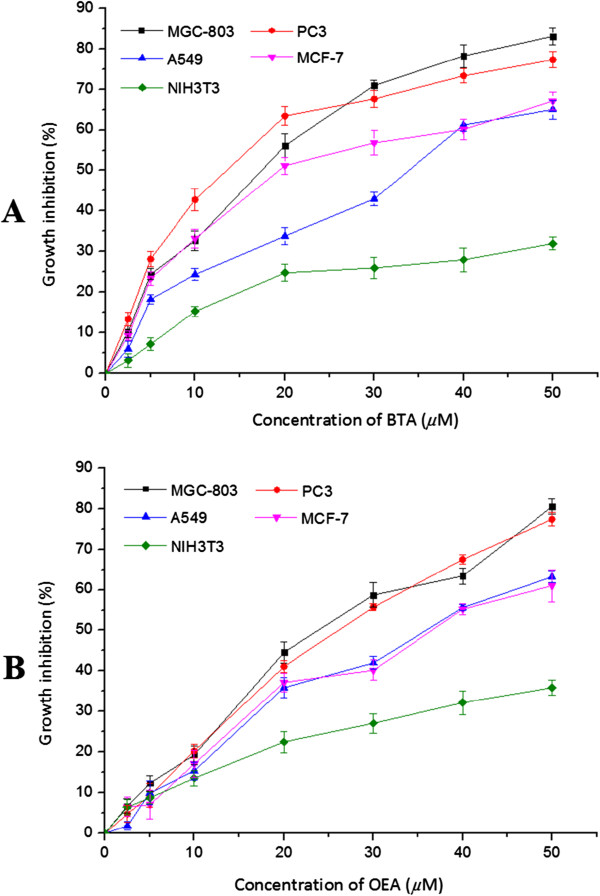
**Effect of BTA and OEA on proliferation of tumor cells.** Data are presented as means ± SD, n = 4

To best of our knowledge, the two compounds, BTA and OEA, were obtained from *Toona sinensis* for the first time*.* It was also found to have the greatest potency against the growth of human cancer cell lines and little toxic effect on NIH3T3 cells among the isolated constituents. Further experiments found that proliferation of these four cancer cells were significantly inhibited by BTA and OEA in a concentration-dependent manner, as shown in Figure 1A and 1B. The IC_50_ values of BTA and OEA on MGC-803 and PC3 cells were determined to be 17.7 *μ*M and 13.6 *μ*M, 26.5 *μ*M and 21.9 *μ*M, respectively, all of which were lower than that on NIH3T3 cells (IC_50_ > 50 *μ*M) by MTT assay. On this occasion, the two compounds were both less toxic on normal cells than on the investigated cancer cell lines and much selective to cancer cells.

Apoptosis is a physiological pattern of cell death characterized by morphological features and extensive DNA fragmentation [34]. Thus, to determine whether the grown inhibitory activities of BTA and OEA were related to the induction of apoptosis, the morphological changes of MGC-803 and PC3 cells were investigated using acridine orange/ethidium bromide (AO/EB) staining and Hoechst 33258 staining, and Terminal deoxynucleotidyl transferase dUTP nick end labeling (TUNEL) assay to confirm cell apoptosis. Moreover, the apoptosis ratios induced by BTA and OEA caused apoptosis in MGC-803 cells were quantitatively assessed by flow cytometry (FCM). Interestingly, whether the cancer cell apoptosis by the two compounds was though the mitochondrial pathway was also studied.

**Figure 2:**
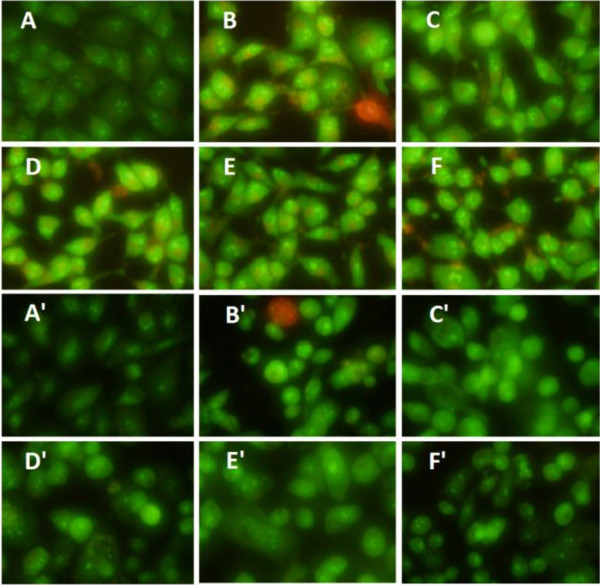
**Results from the AO/EB staining.** For MGC-803 cells group, **A**: negative control; **B**: positive control, treated with HCPT (20 *μ*M) for 48 h; **C**, **D**: treated with BTA (20 *μ*M) for 24, 48 h; **E**, **F**: treated with OEA (20 *μ*M) for 24, 48 h. For PC3 cells group, **A**’: negative control; **B**’: positive control, treated with HCPT (20 *μ*M) for 48 h; **C**’, **D**’: treated with BTA (20 *μ*M) for 24, 48 h; **E**’, **F**’: treated with OEA (20 *μ*M) for 24, 48 h

AO is taken up by both viable and non-viable cells and emits green fluorescence if intercalated into double stranded nucleic acid (DNA), and EB is taken up only by non-viable cells and emits red fluorescence by intercalation into DNA. Thus, live cells have a normal green nucleus, whereas the early apoptotic cells are bright green nucleus with condensed or fragmented chromatin and the late apoptotic cells display condensed and fragmented orange chromatin [35]. With HCPT as positive control, the BTA and OEA at 20 *μ*M for 24, 48 h were detected *via* AO/EB staining. As can be seen in Figure 2, early apoptotic cells with yellow dots and late apoptotic cells with orange dots in MGC-803 and PC3 cell nuclei in positive control, and the cells treated with BTA and OEA had changed. Yellow and orange dots in MGC-803 and PC3 cells showed early and late apoptotic cells, and the appearance of little red cells indicated that BTA and OEA were low cytotoxicity. Therefore, it can be concluded that BTA and OEA could induce apoptosis without any significant cytotoxicity.

**Figure 3:**
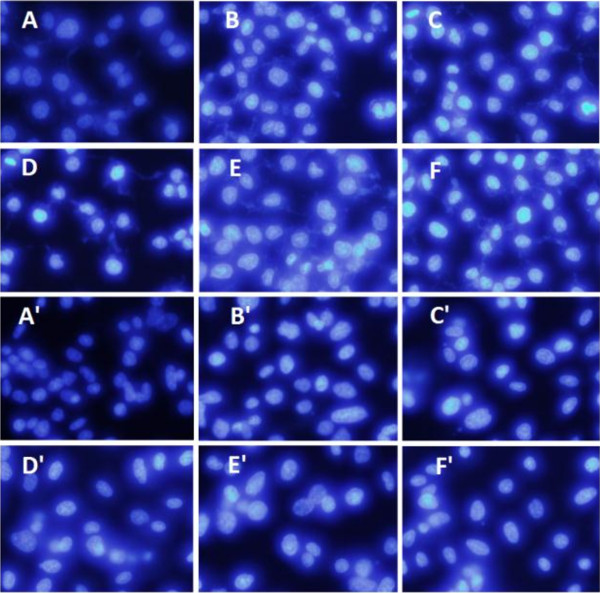
**Results from the Hoechst 33258 staining.** For MGC-803 cells group, **A**: negative control; **B**: positive control, treated with HCPT (20 *μ*M) for 48 h; **C**, **D**: treated with BTA (20 *μ*M) for 24, 48 h; **E**, **F**: treated with OEA (20 *μ*M) for 24, 48 h. For PC3 cells group, **A**’: negative control; **B**’: positive control, treated with HCPT (20 *μ*M) for 48 h; **C**’, **D**’: treated with BTA (20 *μ*M) for 24, 48 h; **E**’, **F**’: treated with OEA (20 *μ*M) for 24, 48 h

Hoechst 33258 staining is used to visualize nuclear changes and apoptotic body formation that are characteristic of apoptosis. And it showed apoptosis in all four types of cells, which were characterized by cytoplasmic and nuclear shrinkage, chromatin condensation and apoptosis body [36]. With HCPT as positive control, the BTA and OEA at 20 *μ*M for 24, 48 h were detected *via* Hoechst 33258 staining. As shown in Figure 3, cells treated with the negative control were normally blue. The cells of the negative group were normal blue. However, the HCPT group appeared compact condensed, and crescent-shaped. The cells exhibited strong blue fluorescence, revealing the typical apoptosis characteristics. The cells treated with BTA and OEA had changed, and cells nuclei appeared to be highly condensed and crescent-shaped. These findings demonstrate that BTA and OEA could induce apoptosis against MGC-803 and PC3 cell lines, consistent with the results for the previous AO/EB double staining.

**Figure 4:**
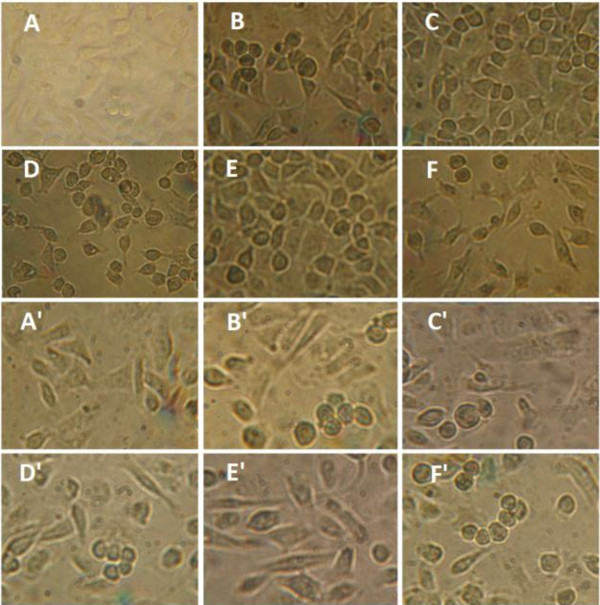
**Results from the TUNEL assay.** For MGC-803 cells group, **A**: negative control; **B**: positive control, treated with HCPT (20 *μ*M) for 48 h; **C**, **D**: treated with BTA (20 *μ*M) for 24, 48 h; **E**, **F**: treated with OEA (20 *μ*M) for 24, 48 h. For PC3 cells group, **A**’: negative control; **B**’: positive control, treated with HCPT (20 *μ*M) for 48 h; **C**’, **D**’: treated with BTA (20 *μ*M) for 24, 48 h; **E**’, **F**’: treated with OEA (20 *μ*M) for 24, 48 h

In addition, TUNEL, one of the popular methods to investigate the apoptosis induction, identified apoptotic cells *in situ via* the detection of DNA fragmentation, due to the degradation of DNA after the activation of Ca/Mg-dependent endonucleases. This DNA cleavage leads to strand breaks within the DNA, and could be identified by terminal deoxynucleotidyl transferase that catalyzed the addition of biotin-dUTP. The biotin-labeled cleavage sites were then detected by reaction with streptavidin-HRP and visualized by diaminobenzidine, as indicated by a brown color [37]. With HCPT as positive control, the BTA and OEA at 20 *μ*M for 24, 48 h were detected *via* TUNEL assay. As shown in Figure 4, the cells treated with BTA, OEA and HCPT appear as brown precipitates. Therefore, it can be further concluded that BTA and OEA could induced apoptosis in MGC-803 and PC3 cells. The results were identical with the previous experiment.

**Figure 5:**
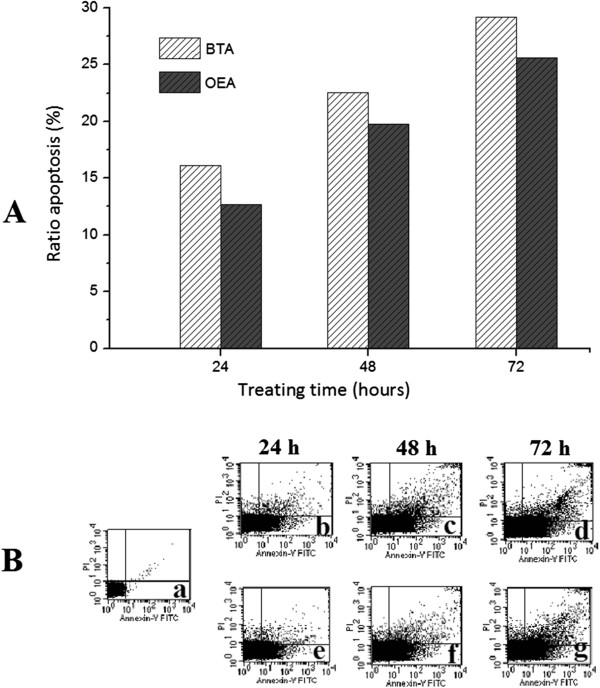
**Flow cytometry analysis. A**: the apoptosis ratios of MGC-803 cells treated with BTA and OEA (20 *μ*M) assessed by flow cytometry. **B**: flow cytometry analysis for apoptosis inducing activities of BTA and OEA on MGC-803 cells, a: control; b, c, and d: treated with BTA (20 *μ*M); e, f, and g: treated with OEA (20 *μ*M)

The apoptosis ratios induced by BTA and OEA in MGC-803 cells were quantitatively assessed by FCM. In early apoptotic cells, phosphatidylserine (PS) which distributed inside the lipid bilayer in the normal cells was transferred from the inside of the cell membrane to the outside. Annexin V, a Ca^2+^ dependent phospholipid-binding protein with a high affinity for PS, was used to detect early apoptotic cells. PI (Propidine Iodide) was a red fluorescent dye and stained cells that had lost membrane integrity. Cells stained with Annexin V-FITC and PI were classified as necrotic cells (the upper left quadrant; Annexin^−^/PI^+^), late apoptotic cells (the upper right quadrant; Annexin^+^/PI^+^), intact cells (the lower left quadrant; Annexin^−^/PI^−^) or early apoptotic cells (the lower right quadrant; Annexin^+^/PI^−^) [38]. As shown in Figure 5A, BTA and OEA could induce apoptosis in MGC-803 cells. Apoptosis ratios (including the early and late apoptosis ratios) for BTA and OEA were obtained after 72 h of treatment at a concentration of 20 *μ*M, with the highest apoptosis ratios being 27.3% and 24.5%, respectively. Furthermore, as shown in Figure 5B, the apoptosis of MGC-803 cells which treated with BTA and OEA increased gradually in a time-dependent manner.

**Figure 6:**
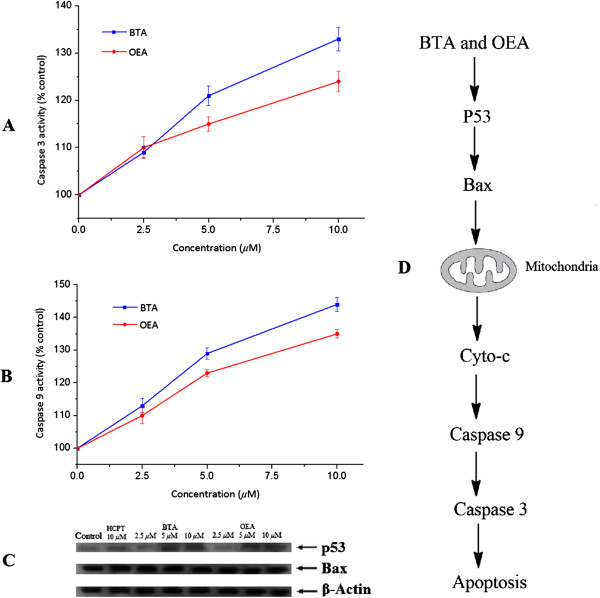
**Levels of caspases, p53, and Bax. A**: activation of caspase 3 in MGC-803 cells; **B**: activation of caspase 9 in MGC-803 cells; **C**: western blot analysis of p53 and Bax in MGC-803 cells; **D**: cell apoptosis was mediated by the mitochondria pathway

p53 could induce apoptosis after DNA damage in cancer cells [39], while the pro-apoptotic bcl-2 family member, Bax was a candidate mediator of p53-induced apoptosis [40]. The bcl-2 family divided into pro-survival members such as Bcl-2, Bcl-XL, Bcl-w, and CED 9 and pro-apoptotic members such as Bax, Bad, and Bid [41]. On this occasion, these opposing family members could heterodimerize and the relative ratio of the pro-survival vs. pro-apoptotic members may determine whether the cell lives or dies [42]. The anti-apoptotic members appear to function by inhibiting the release of cytochrome c from the mitochondria or by inhibiting Apaf-1 directly [43]. Cytochrome c acts as a co-factor with ATP for the activation of Apaf-1 which then activates caspase 9, an “initiator caspase”, and caspase 9 can then in turn activate caspase 3 [44]. As shown in Figure 6A, 6B, and 6C, when MGC-803 cells were treated with BTA and OEA at different concentrations after 12 h, the caspase 3/9, p53, and Bax were activated significantly. Thus, the results revealed that the BTA and OEA could induce mitochondria pathway mediated cell apoptosis in MGC-803 cell line (Figure 6D).

## Conclusions

In conclusion, studies on the chemical constituents from *Toona sinensis*, and their biological activities have assumed significance for the rational development and utilization of this plant. In this study, fifteen compounds were isolated and identified. Meanwhile, the tumor cell growth inhibition effects of these constituents on MGC-803, PC3, A549 and MCF-7 cells were carried out by MTT assay. Among these compounds, BTA and OEA, which were isolated from *Toona sinensis*, showed potent activities on MGC-803 and PC3 cell lines in a dose-dependent manner. The IC_50_ values of BTA and OEA on MGC-803 and PC3 cells were determined to be 17.7 *μ*M and 13.6 *μ*M, 26.5 *μ*M and 21.9 *μ*M, respectively, all of which were lower than that on NIH3T3 cells (IC_50_ > 50 *μ*M). The apoptosis inducing activities of BTA and OEA on MGC-803 and PC3 cell lines were investigated through AO/EB staining, Hoechst 33258 staining, and TUNEL assay. In addition, the apoptosis ratios induced by BTA and OEA caused apoptosis of MGC-803 cells were quantitatively assessed by flow cytometry, with apoptosis ratios of 27.3% and 24.5% after 72 h of treatment at 20 *μ*M, respectively. Interestingly, the BTA and OEA induced cell apoptosis through the mitochondrial pathway in MGC-803 cells. Our findings have implied that BTA and OEA has potential therapeutic value for treatment of cancer.

## Competing interest

The authors declare there are not any competing interests.

## Authors’ contribution

SY designed the experiments and carried out most of the bioassay experiments. QZ and HX took part in the compound structural elucidation and bioassay experiments. ML took part of the bioassay experiments. QZ and WX carried out some structure elucidation experiments. Prof. BS and Prof. SY are the co-corresponding authors for this work. All authors read and approved the final manuscript.

## Supplementary Material

Additional file 1 The extraction and purification process of the compounds from the plant and their NMR data. Click here for file
