# Supplementary material for: Antiproliferative activity and apoptosis-inducing mechanism of constituents from Toona sinensis on human cancer cells
Source: Cancer Cell Int. 2013 Feb 9;13:12. doi: 10.1186/1475-2867-13-12 (PMC3620677; doi:10.1186/1475-2867-13-12)
Supplement: Additional file 1 — The extraction and purification process of the compounds from the plant and their NMR data. [file 1475-2867-13-12-S1.doc]

Additional file 1

# Antiproliferative activity and apoptosis-inducing mechanism of constituents from *Toona sinensis* on human cancer cells

Shengjie Yang, Qi Zhao, Hongmei Xiang, Minjie Liu, Qiuyun Zhang, Wei Xue, Baoan Song^*^ and Song Yang^*^

State-Local Joint Laboratory for Comprehensive Utilization of Biomass, State Key Laboratory Breeding Base of Green Pesticide and Agricultural Bioengineering, Key Laboratory of Green Pesticide and Agricultural Bioengineering, Ministry of Education, Guizhou University, Guiyang 550025, P.R. China

*Corresponding authors. Ctr for R&D of Fine Chemicals, Guizhou University, Huaxi St., Guiyang, China 550025. Tel.: +86 851 829 2171; Fax: +86 851 829 2170.

E-mail addresses: songbaoan60@yahoo.cn, yangstg2003@gmail.com

## Analysis and Instruments

The melting points of the products were determined using an XT-4 binocular microscope (Beijing Tech Instrument Co. Ltd., Beijing, China). Infrared spectra were recorded on a Bruker VECTOR22 spectrometer in KBr disks. ^1^H-NMR and ^13^C-NMR were recorded using a JEOL-ECX500 spectrometer at 22 °C, with tetramethylsilane as the internal standard and CDCl_3_, DMSO-*d*_6_, CD_3_COCD_3_, or CD_3_OD as the solvent. Column chromatography was performed using silica gel (200–300 meshes) (Qingdao Marine Chemistry Co., Qingdao, China) and silica gel H (Qingdao Marine Chemistry Co., Qingdao, China), Sephadex LH-20 (GE Healthcare Bio-Sciences AB, Uppsala, Sweden), HP-20 (Mitsubishi Chemical Corp., Tokyo Met, Japan), and MCI-gel CHP 20P (Mitsubishi Chemical Corp., Toukyu Met, Japan). All other chemicals were of analytical reagent grade and used without further purification.

## Extraction and isolation

Dried roots of *Toona sinensis* (8 kg) were cut into pieces and extracted with 80% EtOH (3×40 L) under reflux, 6 h for the first time, 3 h for the second time and 1 h for the last time. The combined EtOH extracts were evaporated to dryness to yield a dried EtOH extract (2.5 kg). The extract was suspended in water and then extracted with petroleum ether (10 L×1 times), ethyl acetate (10 L×4 times) and *n*-BuOH (10 L×5 times) respectively. Petroleum ether extract (26 g), ethyl acetate extract (250 g), and *n*-BuOH extract (525 g) were obtained.

The viscous dark mass of the ethyl acetate extract (110 g) was subjected to CC on silica gel (200-300 mesh) eluted with a gradient of petroleum ether-EtOAc (20/1, 10/1, 5/1, 2/1 and 1/1, v/v), and finally a mixture of chloroform and methanol (10/1, 5/1 and 1/1, v/v, respectively) to get 10 fractions, namely, fractions 1-10. The fractions were monitored by TLC. Fraction 4 (15 g) was applied to a silica gel (200-300 mesh) column eluted with petroleum ether-EtOAc (10:1 to 1:1) to yield *β*-sitosterol (**1**, 42 mg), and yield 2 fractions, namely, fractions A and B. Fraction A was purified using PTLC to yield *α*-amyrin (**2**, 25 mg). Fraction B was recrystallized with CHCl_3_-MeOH (20:1) to afford daucosterol (**3**, 29 mg). Fraction 5 (2.2 g) was applied to a silica gel H column eluted with petroleum ether-EtOAc (10:1-1:1) to yield a yellow powder (867 mg), which was purified using a Sephadex LH-20 column eluted with CHCl_3_-MeOH (1:1) to yield quercetin (**4**, 25 mg) and (+)-catechin hydrate (**5**, mg). Fraction 6 (2.8 g) was separated using HP-20 resineluted with 50%-100% MeOH-H_2_O and purified using a silica gel column to yield (-)-epicatchin (**6**, 14 mg), kampferol (**7**, 20 mg) and 3-oxours-12-en-28-oic acid (**8**, 26 mg). Fraction 7 (4.6 g) was separated using silica gel (300-400 mesh) eluted with petroleum ether-EtOAc (10:1) to yield ursolic acid (**9**, 27 mg) and betulonic acid (**10**, 31 mg). Fraction 8 was decolorized and separated on MCI gel to yield gallic acid (**11**, 36 mg) and sucrose (**12**, 41 mg). Fraction 9 was purified using a sephadex LH-20 column eluted with CHCl_3_-MeOH (1:1) to yield white powder (943 mg). The white powder was purified using a silica gel (300-400 mesh) to yield betulinic acid (**13**, 21 mg), myricitrin (**14**, 18mg), and caffeic acid (**15**, 16 mg).

All spots on TLC were visualized by heating silica gel plates sprayed with 10% phosphomolybdic acid hydrate in EtOH and 1% FeCl_3_ in EtOH.

## Data of compounds isolated from *Toona sinensis*

*β*-Sitosterol (**1**), white needle; mp 141-143 ^o^C; IR (KBr, cm^-1^) ν_max_ 3419, 1639, 1379, 1055; ^1^H NMR (500 MHz, CDCl_3_) δ: 5.35 (1H, brs, H-6), 3.51 (1H, m, H-3), 1.04 (3H, s, CH_3_), 0.90 (3H, s, CH_3_), 0.86 (3H, s, CH_3_), 0.84 (3H, s, CH_3_), 0.80 (3H, s, CH_3_), 0.63 (3H, s, CH_3_); ^13^C NMR (125 MHz, CDCl_3_) δ: 140.8 (C-5), 121.8 (C-6), 71.9 (C-3), 56.8 (C-14), 56.1 (C-17), 50.1 (C-9), 45.9 (C-24), 42.3 (C-13), 42.3 (C-4), 39.8 (C-12), 37.3 (C-1), 36.6 (C-10), 36.2 (C-20), 34.0 (C-22), 31.7 (C-2), 29.2 (C-25), 28.3 (C-16), 26.1 (C-23), 24.4 (C-15), 23.1 (C-28), 21.2 (C-11), 19.9 (C-26), 19.5 (C-19), 19.1 (C-27), 18.8 (C-21), 12.0 (C-29), 11.9 (C-18). The above data were identical to the literature data [1].

*α*-Amyrin (**2**), white powder; mp 168-170 ^o^C; IR (KBr, cm^-1^) ν_max_ 3312, 1502, 1041; ^1^H NMR (500 MHz, CDCl_3_) δ: 5.25 (1H, t, *J* = 3.5 Hz, H-12), 3.20 (1H, brs, H-3), 1.08 (3H, s, CH_3_), 0.98 (3H, s, CH_3_), 0.87 (3H, s, CH_3_), 0.85 (3H, s, CH_3_), 0.79 (3H, s, CH_3_); ^13^C NMR (125 MHz, CDCl_3_) δ: 138.0 (C-13), 125.9 (C-12), 79.1 (C-3), 55.3 (C-18), 52.8 (C-5), 47.6 (C-9), 42.1 (C-14), 40.0 (C-22), 39.1 (C-8), 38.9 (C-1), 38.8 (C-19), 38.7 (C-20), 37.1 (C-4), 36.8 (C-10), 33.1 (C-17), 30.7 (C-7), 29.8 (C-21), 27.3 (C-28), 24.3 (C-16), 23.6 (C-11), 23.4 (C-27), 21.2 (C-30), 18.4 (C-6), 17.2 (C-29), 17.0 (C-26), 15.6 (C-24),15.5 (C-25). The above data were identical to the literature data [2].

Daucosterol (**3**), white powder; mp 282-284 ^o^C; IR (KBr, cm^-1^) ν_max_ 3382, 2860, 1450, 1060; ^1^H NMR (500 MHz, DMSO-*d_6_*) δ: 5.28 (1H, s, H-6), 4.17 (1H, m, H-3), 0.95 (3H, s, CH_3_), 0.92 (3H, s, CH_3_), 0.87 (3H, s, CH_3_), 0.86 (3H, s, CH_3_), 0.85 (3H, s, CH_3_), 0.64 (3H, d, *J* = 10.5 Hz, CH_3_); ^13^C NMR (125 MHz, DMSO-*d_6_*) δ: 140.9 (C-5), 121.7 (C-6), 101.2 (C-1’), 77.4 (C-3’), 77.2 (C-3), 77.2 (C-5’), 73.9 (C-2’), 70.6 (C-4’), 61.6 (C-6’), 56.7 (C-14), 55.9 (C-17), 50.1 (C-9), 45.6 (C-24), 42.3 (C-13), 38.8 (C-4), 37.3 (C-1), 37.0 (C-20), 36.7 (C-10), 33.8 (C-22), 31.9 (C-8), 31.9 (C-7), 29.7 (C-2), 29.2 (C-25), 28.3 (C-16), 25.9 (C-23), 24.4 (C-15), 23.1 (C-28), 21.1 (C-11), 20.2 (C-27), 19.6 (C-26), 19.4 (C-19), 19.1 (C-21), 12.3 (C-18), 12.2 (C-29). The above data were identical to the literature data [3].

Quercetin (**4**), yellow powder; mp 283-285 ^o^C; IR (KBr, cm^-1^) ν_max_ 3339, 1650, 1620, 1510; ^1^H NMR (500 MHz, CD_3_OD) δ: 7.79 (1H, d, *J* = 1.5 Hz, H-2’), 7.59 (1H, dd, *J* = 2.5 Hz, 5 Hz, H-6), 6.77 (1H, d, *J* =3 Hz, H-5’), 6.30 (1H, d, *J* = 4.5 Hz, H-8), 6.11 (1H, d, *J* = 2.5 Hz, H-6); ^13^C NMR (125 MHz, CD_3_OD) δ: 175.8 (C-4), 163.8 (C-7), 161.5 (C-5), 156.9 (C-9), 147.8 (C-2), 147.6 (C-3’), 145.2 (C-4’), 123.0 (C-1’), 121.0 (C-6’), 114.8 (C-2’), 114.6 (C-5’), 103.5 (C-10), 97.9 (C-6), 93.2 (C-8). The above data were identical to the literature data [4].

(+)-Catechin hydrate (**5**), white powder; mp 134-136 ^o^C; IR (KBr, cm^-1^) ν_max_ 3350, 1635, 1511; ^1^H-NMR (500 MHz, CD_3_OD) δ: 6.81 (1H, d, *J* = 1 Hz, H-2’), 6.72 (1H, d, *J* = 8 Hz, H-6’), 6.70 (1H, d, *J* = 1.5 Hz, H-5), 5.90 (1H, d, *J* = 3 Hz, H-8), 5.82 (1H, d, *J* = 2 Hz, H-6), 4.54 (1H, d, *J* = 7 Hz, H-2), 2.83 (1H, dd, *J* = 5.5, 15 Hz, H-3), 2.47 (1H, dd, *J* = 10, 14 Hz, H-4); ^13^C-NMR (125 MHz, CD_3_OD) δ: 156.5 (C-7), 156.2 (C-9), 155.6 (C-5), 144.9 (C-4’), 144.9 (C-3’), 130.8 (C-1’), 118.7 (C-6’), 114.7 (C-5’), 113.9 (C-2’), 99.5 (C-10), 94.9 (C-6), 94.1 (C-8), 81.5 (C-2), 67.4 (C-3), 27.2 (C-4). The above data were identical to the literature data [5].

(-)-Epicatchin (**6**), white powder; mp 222-224 ^o^C; IR (KBr, cm^-1^) ν_max_ 3440, 1622, 1461, 1289; ^1^H NMR (500 MHz, CD_3_OD) δ: 6.79 (1H, d, *J* = 5 Hz, H-6’), 6.75 (1H, d, *J* = 2 Hz, H-2’), 6.70 (1H, d, *J* = 1.5 Hz, H-5), 5.93 (1H, d, *J* = 3 Hz, H-8), 5.84 (1H, d, *J* = 1.5 Hz, H-6), 4.80 (1H, s, H-2), 2.85 (1H, dd, *J* = 7.5 Hz, 12.5 Hz, H-3), 2.48 (1H, dd, *J* = 10, 12 Hz, H-4); ^13^C NMR (125 MHz, CD_3_OD) δ: 156.8 (C-9), 156.2 (C-7), 155.5 (C-5), 144.8 (C-3’), 143.9 (C-4’), 130.9 (C-1’), 118.0 (C-6’), 114.7 (C-2’), 113.9 (C-5’), 98.7 (C-10), 95.0 (C-6), 94.5 (C-8), 78.5 (C-2), 66.1 (C-3), 27.9 (C-4). The above data were identical to the literature data [6].

Kampferol (**7**), yellow powder; mp 279-281 ^o^C; IR (KBr, cm^-1^) ν_max_ 3409, 1649, 1511; ^1^H NMR (500 MHz, CD_3_OD) δ: 7.98 (2H, d, *J* = 1.5 Hz, H-2’, H-6’), 6.88 (2H, d, *J* = 4 Hz, H-3’, H-5’), 6.35 (1H, d, *J* = 1 Hz, H-8), 6.11 (1H, d, *J* = 2 Hz, H-6). ^13^C NMR (125 MHz, CD_3_OD) δ: 175.8 (C-4), 164.1 (C-7), 161.2 (C-9), 159.2 (C-4’), 156.9 (C-5), 146.2 (C-2), 135.8 (C-6’), 129.6 (C-2’), 122.5 (C-1’), 115.5 (C-5’), 115.4 (C-3’), 103.3 (C-10), 98.3 (C-6), 93.7 (C-8). The above data were identical to the literature data [7].

3-Oxours-12-en-28-oic acid (**8**), white powder; mp 261-262 ^o^C；IR (KBr, cm^-1^) ν_max_ 3438, 2930, 2864, 1638; ^1^H NMR (500 MHz, CD_3_OD) δ: 5.25 (1H, m, H-12), 2.18 (1H, d, *J* = 9.5 Hz, H-18), 1.24 (3H, s, CH_3_), 1.07 (3H, s, CH_3_), 1.04 (3H, s, CH_3_),1.01 (3H, s, CH_3_), 0.84 (3H, s, CH_3_), 0.79 (3H, s, CH_3_); ^13^C NMR (125 MHz, CD_3_OD) δ: 217.9 (C-3), 183.7 (C-28), 138.1 (C-13), 125.7 (C-12), 55.3(C-5), 52.7 (C-18), 48.1 (C-17), 47.5 (C-4), 46.8 (C-9), 42.1 (C-14), 39.5 (C-8), 39.3 (C-1), 39.1 (C-19), 38.9 (C-20), 36.7 (C-22), 36.7 (C-10), 34.2 (C-2), 32.5 (C-7), 30.7 (C-21), 28.0 (C-15), 26.6 (C-23), 24.1 (C-16), 23.6 (C-27), 23.5 (C-11), 21.5 (C-24), 21.2 (C-30), 19.7 (C-6), 17.1 (C-29), 17.0 (C-26), 15.3 (C-25) [8].

Ursolic acid (**9**), white powder; mp 253-255 ^o^C；IR (KBr, cm^-1^) ν_max_ 3442, 2918, 2856, 1688, 1420, 1011; ^1^H NMR (500 MHz, CD_3_OD) δ: 5.22 (1H, m, H-12), 3.14 (1H, m, H-3), 2.18 (1H, d, *J* = 10.5 Hz, H-18), 1.12 (3H, s, CH_3_), 0.96 (3H, s, CH_3_), 0.89 (3H, s, CH_3_), 0.84 (3H, s, CH_3_), 0.77 (3H, s, CH_3_); ^13^C NMR (125 MHz, CD_3_OD) δ: 180.1 (C-28), 138.3 (C-1), 125.6 (C-2), 78.4 (C-8), 55.4 (C-9), 53.1 (C-18), 41.9 (C-10), 39.5 (C-14), 39.1 (C-19), 39.0 (C-9), 38.7 (C-20), 38.5 (C-13), 36.7 (C-12), 36.8 (C-5), 33.0 (C-22), 30.4 (C-6), 27.8 (C-16), 27.4 (C-7), 26.5 (C-15), 24.0 (C-21), 23.0 (C-3), 22.7 (C-11), 20.2 (C-25), 18.1 (C-26), 16.4 (C-24), 16.3 (C-23), 15.0 (C-30), 14.6 (C-29). The above data were identical to the literature data [9].

Betulonic acid (**10**), white powder; mp 291-293 ^o^C; IR (KBr, cm^-1^) ν_max_ 3440, 2945, 1689, 1680; ^1^H NMR (500 MHz, CDCl_3_) δ: 4.71 (1H, s, H-29), 4.58 (1H, s, H-29), 3.01 (3H, m, H-2, 19), 1.71 (3H, s, CH_3_), 1.10 (3H, s, CH_3_), 0.99 (3H, s, CH_3_), 0.95 (3H, s, CH_3_), 0.93 (3H, s, CH_3_); ^13^C NMR (125 MHz, CDCl_3_) δ: 219.5 (C-3), 181.8 (C-28), 150.4 (C-20), 109.7 (C-29), 55.9 (C-17), 54.8 (C-5), 49.8 (C-9), 49.5 (C-18), 47.4 (C-4), 47.0 (C-19), 42.5 (C-14), 40.6 (C-8), 39.7 (C-1), 38.6 (C-13), 37.2 (C-22), 36.8 (C-10), 34.2 (C-2), 33.7 (C-7), 32.2 (C-16), 30.7 (C-21), 29.7 (C-15), 26.8 (C-23), 25.6 (C-12), 21.5 (C-11),21.3 (C-24), 19.5 (C-6), 19.4 (C-30), 16.1 (C-26), 15.8 (C-25), 14.5 (C-27). The above data were identical to the literature data [10].

Gallic acid (**11**), white powder; mp 258-260 ^o^C; IR (KBr, cm^-1^) ν_max_ 3401, 3259, 2363, 1611, 1230, 1029; ^1^H NMR (500 MHz, CD_3_OD) δ: 6.88 (2H, s, PhH); ^13^C NMR (125 MHz, CD_3_OD) δ: 169.1 (C=O), 145.6 (C-3, 5), 138.2 (C-4), 120.6 (C-1), 108.9 (C-2, 6). The above data were identical to the literature data [11].

Sucrose (**12**), colorless cubic crystal; mp 188-190 ^o^C; ^1^H NMR (500 MHz, D_2_O) δ: 5.26 (1H, d, *J* = 5 Hz, H-1), 4.07 (1H, d, *J* = 10 Hz, H-2); ^13^C NMR (125 MHz, D_2_O) δ: 103.6 (C-2′), 92.1 (C-1), 81.3 (C-3′), 76.3 (C-4′), 73.9 (C-5′), 72.5 (C-2), 72.3 (C-3), 71.0 (C-5), 69.1 (C-4), 62.3 (C-1′), 61.2 (C-6′), 60.0 (C-6). The above data were identical to the literature data [12].

Betulinic acid (**13**), white powder; mp 238-340 ^o^C; IR (KBr, cm^-1^) ν_max_ 3440, 2925, 1690, 1460, 1039; 1H NMR (500 MHz, CD_3_OD) δ: 3.20 (1H, m, H-3), 0.98 (3H, s, CH_3_), 0.95 (3H, s, CH_3_), 0.93 (3H, s, CH_3_), 0.84 (3H, s, CH_3_), 0.74 (3H, s, CH_3_); ^13^C NMR (125 MHz, CD_3_OD) δ: 178.9 (C-28), 150.7 (C-20), 109.4 (C-29), 78. 4 (C-3), 55.5 (C-17), 51.0 (C-5), 50.9 (C-9), 48.9 (C-19), 45.0 (C-18), 42.4 (C-14), 40.7 (C-8), 38.9 (C-4), 38.9 (C-1), 38.5 (C-13), 37.0 (C-10), 36.9 (C-22), 34.3 (C-7), 32.2 (C-16), 30.7 (C-21), 29.9 (C-15), 25.6 (C-2),26.9 (C-23), 22.7 (C-12), 20.9(C-11), 18.4 (C-30), 18.3 (C-6), 15.5 (C-25), 15.4 (C-26), 14.7 (C-27), 13.7 (C-24). The above data were identical to the literature data [13].

Myricitrin (**14**), yellow needle crystal; mp 167-169 ^o^C; IR (KBr, cm^-1^) ν_max_ 3300, 2948, 1656, 1511, 1448; ^1^ H NMR (500 Hz, CD_3_OD) δ: 6.92 (2H, s, H-2’, 6’), 6.34 (1H, d, *J* = 2 Hz, H-8), 6.18 (1H, d, *J* = 2 Hz), 5.29 (1H, s, rha H-1), 0.95 (3H, d, *J* = 6.5 Hz, CH_3_); ^13^ C NMR (125 Hz, CD_3_OD) δ: 178.3 (C-4), 164.5 (C-7), 161.9 (C-5), 158.1 (C-2), 157.2 (C-9), 145.5 (C-3’, 5’), 136.5 (C-4’), 134.9 (C-3), 120.6 (C-1’), 108.2 (C-2’, 6’), 104.5 (C-10), 102.3 (rha C-1), 98.4 (C-6), 93.3 (C-8), 72.0 (rha C-4), 70.8 (rha C-2), 70.7 (rha C-3), 70.5 (rha C-5), 16.3 (rha C-6). The above data were identical to the literature data [14].

Caffeic acid (**15**), yellow powder; mp 178-180^o^C; IR (KBr, cm^-1^) ν_max_ 3451, 1640, 1611, 1518; ^1^ H NMR (500 Hz, CD_3_COCD_3_) δ: 7.52 (1H, d, *J* = 14.5 Hz, H-7), 7.00 (1H, s, H-2), 6.84 (1H, d, *J* = 7.5 Hz, H-5), 6.24 (1H, d, *J* = 15 Hz, H-8); ^13^ C NMR (125 Hz, CD_3_COCD_3_) δ: 167.3 (C-9), 147.9 (C-4), 145.5 (C-3), 145.1 (C-7), 126.8 (C-1), 121.6 (C-8), 115.5 (C-2), 114.9 (C-5), 114.3 (C-6). The above data were identical to the literature data [15].

## References

1. Xie XY, Gong JY, Wang LY, Zhang ZJ, Yu CY: **Chemical constituents of the root of *Rosa davurica pall*.** *Lishizhen Med Mater Medica Res* 2009, **20**:366-367.
2. Robinson A, Sunkara Y, Suresh BK, Roa JM, Madhavendra SS: **Isolation of *α*-amyrin eicosanoate, a triterpenoid from the roots of *Saussurea lappa* clarke-differential solubility as an aid.** *J Pharm Sci Tech* 2012, **2**:207-212.
3. Kurkin VA, Kurkina TV, Zapesochnaya GG, Avdeeva EV, Bogolyubova ZV, Vandyshev VV, Chikina IY: **Chemical investigation of the herbage of *Melissa officinalis*.** *Chem Nat Compd* 1995, **31**:266-267.
4. Mao YW, Tseng HW, Liang WL, Chen IS, Chen ST: **Anti-inflammatory and free radial scavenging activities of the constituents isolated from *Machliuszuihoensis*.** *Molecules* 2011, **16**:9451-9466.
5. Almahy HA, Alhassan NI: **Studies on the chemical constituents of the leaves of *Ficus bengalensis* and their antimicrobial activity.** *J Sci Technol* 2011, **12**:111-116.
6. Galotta ALQA, Boaventura MAD, Lima LARS: **Antioxidant and cytotoxic activities of ‘açaí’ (*Euterpe precatoria* Mart.)**. *Quim Nova* 2008, **31**:1427-1430.
7. Hadizadeh F, Khalili N, Hosseinzadeh H, Khair-Aldine R: **Kaemferol from *Saffron petals.*** *Iran J Pharm Res* 2003, **2**:251-252.
8. Wang QH, Li ZY, Shen Y, Lin HW, Shu W, Zhou JF: S**tudies on triterpenoids from *Potentilla chinensis*.** *China J Chin Mater Medica* 2006, **31**:1434-1436.
9. Masataka M, Yukari I, Kinuko I, Junko K: **New isoflavones from Belamcandae rhizome.** *J Nat Med* 2007, **61**:329.
10. Zhang HJ, Tan GT, Hoang VD, Hung NV, Cuong NM: **Natural Anti-HIV Agents. Part IV. Anti-HIV Constituents from *Vatica cinerea*.** *J Nat Prod* 2003, **2**:263-268.
11. Eldahshan OA: **Isolation and structure elucidation of phenolic compounds of carob leaves grown in Egypt.** *Curr Res J Biol Sci* 2011, **3**:52-55.
12. Zhou L, Wang N, Miao F, Zhao HS, Tian P: **Chemical constituents of *Gentiana apiata* N. E. Br.** *Chin J Org Chem* 2004, **24**:1249-1252.
13. Elvira E, Kovac B, Kemal D, Zdenka K, Emin S: **Identification and isolation of pharmacologically active triterpenes in *Betuale cortex*, *Betula pendula* Roth., Betulaceae.** *Bosnian J Basic Med* 2009, **9**:31-38.
14. Manguro LOA, Ugi I, Lemmen P: **Flavonol glycosides from the leaves of *Embelia keniensis*.** *J Chin Chem Soc* 2005, **52**:201-208.
15. Bhatt B: **Chemical constituents of *Solanum xanthocarpum*.** *J Chem Pharm Res* 2011, **3**:176-181.
